# Cardiovascular Complications of HIV-Associated Immune Dysfunction

**DOI:** 10.1155/2015/302638

**Published:** 2015-01-11

**Authors:** Akram M. Zaaqoq, Faisal A. Khasawneh, Roger D. Smalligan

**Affiliations:** ^1^Department of Internal Medicine, Texas Tech University Health Sciences Center, Amarillo, TX 79106, USA; ^2^Division of Pulmonary and Critical Care Medicine, Department of Internal Medicine, Texas Tech University Health Sciences Center, Amarillo, TX 79106, USA; ^3^Division of Infectious Diseases, Department of Internal Medicine, Texas Tech University Health Sciences Center, Amarillo, TX 79106, USA

## Abstract

Prolonged survival in HIV infection is accompanied by an increased frequency of non-HIV-related comorbidities. It is suggested that cardiovascular diseases (CVD) occur earlier among HIV-positive patients compared with HIV-negative patients, and at a higher rate. Several factors have been proposed which can be categorized into traditional and nontraditional risk factors. Immune dysfunction is a nontraditional risk factor that contributes significantly to cardiovascular pathology. Markers of inflammation are elevated in HIV-infected patients, and elevations in markers such as high-sensitivity C-reactive protein, D-dimer, and interleukin-6 (IL-6) have been associated with increased risk for cardiovascular disease. However, the data currently suggest the most practical advice is to start antiretroviral therapy early and to manage traditional risk factors for CVD aggressively. A better understanding of the mechanisms of CVD in this population and further efforts to modify chronic inflammation remain an important research area.

## 1. Introduction

The reduction of human immunodeficiency virus- (HIV-) related deaths by introduction of antiretroviral therapy (ART) [1] has been challenged by increasing incidence of non-HIV-related mortality [2] that is mainly attributed to cardiovascular diseases [3]. Multiple studies suggest increased risk of cardiovascular disease (CVD) in HIV-infected versus non-HIV-infected patients [4–6]. Although traditional risk factors such as advanced age, smoking, and dyslipidemia [7] have contributed significantly to CVD, nontraditional risk factors such as immune dysfunction have been accused [8]. Through a focused literature search, this review aims to shed light on the cardiovascular complications of persistent immune dysfunction in HIV patients receiving ART as a public health concern and potential preventive strategies to reduce its impact.

## 2. The Burden of Cardiovascular Diseases in HIV Population

Globally, an estimated 35.3 (32.2–38.8) million people were living with HIV in 2012 [9]. More than 95% of HIV infections are in developing countries, two-thirds of them in sub-Saharan Africa [9]. In 2012, 65% of the target group has access to ART; it is up from 54% at the end of 2011 [10]. With increasing access to ART, the life expectancy of HIV-infected individuals is improving. Consequently, mortality from non-HIV-related illness is increasing. Despite the demographic differences of HIV patients between developed and developing countries, CVD remains a major cause of non-HIV-related mortality.

In developed countries, about 9–20% of HIV-positive patients have moderate to high 10-year risk of myocardial infarction (MI) [11, 12]. It is estimated that, by 2015, 50% of HIV-positive patients in the United States of America will be over the age of 50 [13]. Studies have shown that aging HIV-infected patients exhibit significantly increased rates of CVD, including coronary artery disease, MI, and peripheral arterial diseases [4]. Also, traditional risk factors such as smoking, HIV-associated lipodystrophy syndrome (HALS), diabetes mellitus, and hypertension are common among HIV-positive patients [14]. Whereas national estimates indicate that approximately 21% of the adult population smokes [15], the prevalence of active smoking in HIV-positive individuals ranged from 40 to 84% in various studies [16, 17]. Compared with nonsmokers, smokers have a twofold or greater increased risk of CVD [18]. 9–83% of HIV-infected patients suffer from HALS [19]. It represents morphological (lipoatrophy, lipohypertrophy) and metabolic changes in ART treated patients. Patients on ART are exposed to alterations of cholesterol and triglyceride profiles associated with increased risk of atherogenic progression and CVD [19, 20].

In developing countries, about 20% of the daily deaths due to HIV/AIDS are attributed to CVD [21]. This is complicated by rapid epidemiological transition promoted by prolonged survival of HIV-infected individuals, urbanization, and nutritional transition. The percentage of HIV-infected individuals over the age of 50 in South Africa is now higher than in the 15–24-year-old age group [22]. Also this is further increased by the growing number of HIV survivors, now estimated at 5.8% of the population older than 50 years [22]. Urbanization and dietary and lifestyle changes result in conditions such as excessive weight gain, dyslipidemia, and hypertension to become prominent. The rapid epidemiological transition compresses the time available to adopt new strategies and impacts the economy of these countries. In 2010, the total cost of major CVD in the World Health Organization (WHO), Africa subregion, was estimated to be $11.6 billion, including $4.7 billion due to loss of productivity [23]. Therefore, the rapidly increasing CVD in developing countries, with unmatched growth in economy and wealth, will quickly shift these conditions beyond the coping capacities of countries.

## 3. Immune Dysfunction as Nontraditional Risk Factor for CVD

In addition to previously mentioned traditional risk factors for CVD, HIV-induced immune dysfunction might partially explain the increased risk of CVD (Table 1). HIV-induced inflammation might explain the increased risk of CVD in part; particularly it is well established that inflammation is a major factor in the development of atherosclerosis in the general population [24]. Inflammation as a complex biological process represents interplay of multiple cellular and inflammatory mediators that are affected by both HIV and ART [5, 14, 25–27].

## 4. CD4^+^ T-Cells

**Figure 1:**
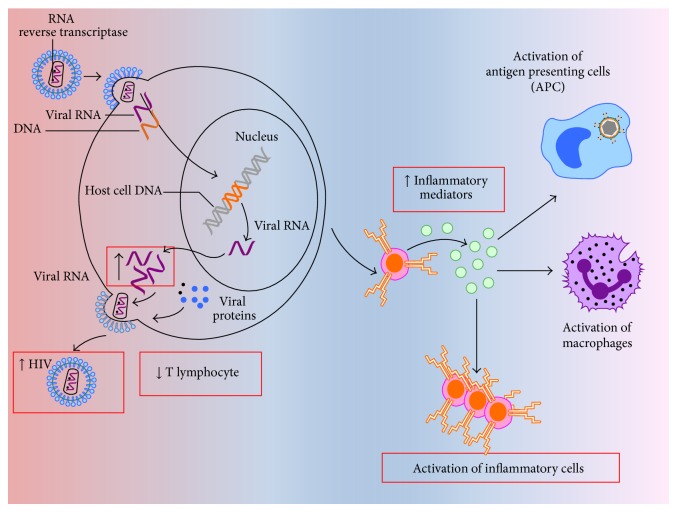
Nontraditional risk factors for cardiovascular diseases in human immunodeficiency virus- (HIV-) infected patients. HIV infection is associated with consumption of CD4^+^ T-cells due to viral replication as indicated by increased viral RNA load (left panel). Subsequently, the increased production of inflammatory mediators such as interleukin-6 (IL-6) indicates a status of dysregulated immune response which precipitates cardiovascular pathology (right panel).

The association between CD4^+^ cell count and CVD has been reported by multiple studies. Although in Data Collection on Adverse Events of Anti-HIV Drugs (D:A:D) study the CD4^+^ cell count below 500 cells/*μ*L was associated with increased risk of non-AIDS-related deaths [28], HIV Outpatient Study (HOPS) cohort reported 28% increased risk of CVD in patients with CD4^+^ count <500 cells/*μ*L, regardless of the class of ART used [29]. In addition, failure to restore a normal peripheral CD4^+^ cell count is associated with an increased risk of morbidity and mortality of CVD [30]. Interestingly, a significant subset of patients who delay therapy until their CD4^+^ cell count is <200 cells/*μ*L may not achieve a normal CD4^+^ cell count, even after >10 years of otherwise effective therapy [31]. These individuals likely remain at risk for developing significant CVD (Figure 1).

## 5. Inflammatory Mediators

Elevated C-reactive protein (CRP), interleukin-6 (IL-6), and D-dimer are predictors of CVD events in the general population [32]. However, the associations between IL-6 and D-dimer levels in HIV-positive individuals with all-cause mortality were much stronger than in studies of non-HIV-infected populations that usually focused on CVD morbidity and mortality [25]. Strategies for Management of Antiretroviral Therapy (SMART) study showed higher levels of the inflammatory or coagulation markers such as high sensitivity CRP, IL-6, D-dimer, and cystatin C in patients with treated HIV disease than in uninfected control subjects. Also it was successfully reported that, one month after stopping treatment, HIV RNA levels were correlated with increases in D-dimer and IL-6 levels and were subsequently associated with an increased risk of all-cause mortality [25]. Furthermore, the Study of Fat Redistribution and Metabolic Change in HIV Infection (FRAM) showed an increase in serum fibrinogen level compared with HIV-negative patients which contributes to increased risk of atherosclerosis in HIV-infected patients [33] (Figure 1).

The underlying mechanism of immune activation is poorly understood; however, multiple studies investigated the potential causes.* HIV replication* below the clinically detectable levels might contribute to persistent immune activation [34]. However, ART intensification trials did not show consistent results which eliminate the role of HIV replication in persistent immune activation [35].* Microbial translocation* as facilitated by HIV-induced depletion of CD4 T-cells from the gut-associated lymphoid tissue and intestinal barrier dysfunction has been proposed as potential cause for persistent immune activation [36, 37]. Even after initiation of ART, microbial translocation does not normalize and continues to be associated with T-cell activation [38]. Majority of HIV-infected individuals are prone to* coinfection* with subsequent immune activation [39]. Cytomegalovirus (CMV) coinfection is highly prevalent in the setting of HIV infection and elicits CMV-specific T-cell responses in HIV-infected individuals [40].

In a study conducted on 49 HIV-infected children with evidence of cardiomyopathy, giving intravenous immunoglobulin (IVIG) was associated with improvement of left ventricular (LV) structure and function as demonstrated by serial echocardiograms [41]. Although the actual mechanism of action of IVIG remains unclear, immunomodulation is proposed as a potential theory. IVIG has been shown to inhibit the production of TNF-*α* via downregulation in at least one study [42].

## 6. Antiretroviral Therapy and Immune Dysfunction

The increasing rates of dyslipidemia and other metabolic changes among HIV-positive patients receiving ART have led to many studies investigating the link between ART use and CVD. In 2003, the D:A:D study demonstrated 26% relative risk increase in rate of MI per year of exposure during the first 4–6 years of use [43]. In a subsequent study, the D:A:D group demonstrated that the rate of MI was 1.53 per 1000 person-years among patients not exposed to protease inhibitors (PIs) and 6.01 per 1000 person-years for patients exposed to PIs for more than 6 years [28]. After adjustment for exposure to the other drug classes and established cardiovascular risk factors (excluding lipid levels), the relative rate of MI per year of PIs exposure was 1.16. The conclusion of the study is that increased exposure to PIs is associated with an increased risk of myocardial infarction, which is partly explained by dyslipidemia.

In an earlier HOPS study, the investigators found an increased risk of MI in patients receiving a PI compared with those who were not. A multivariate statistical analysis showed that PI use was still strongly, although not significantly, associated with the incidence of MI [44]. In a study assessing carotid intima-media thickness (IMT), the investigators found premature atherosclerosis that correlated with usual risk factors, but also with PIs, especially that of lopinavir [45]. The D:A:D study demonstrated that the effect of PIs depends on the time of exposure, hence the need for long follow-up period to detect the actual MI risk [28]. In HOPS study, the exclusion of dyslipidemia made the interpretation of the differences insignificant, although it might reflect casual association between PIs and MI [44].

There are inconsistencies in the literature regarding the risk of MI associated with abacavir usage. Although multiple observational studies point towards an increase in MI risk, the evidence is not consistent [46–51]. However, it is known that even well-conducted observational studies are subject to bias. For instance, in the D:A:D study most patients were not naïve to antiretroviral therapy when enrolled; therefore there was a degree of selection bias which could have affected the patients' survival [52]. On the other hand, three meta-analyses of randomized clinical trials reported no evidence of an association between abacavir use and MI [53–55]. Nevertheless, in a study conducted to evaluate the inflammatory mediators in patients receiving abacavir, there was an associated induction of proinflammatory mediators [CD4 ligand, interleukin-8 (IL-8), and lymphotoxin alpha (LTA)] [56] and these findings might support the concept of a potential MI risk.

## 7. Prevention of Cardiovascular Complications in HIV Patients

Despite the available data on immune dysfunction in HIV-infected patients, it is still unclear how patients should be treated on the basis of this information or whether these markers should be used to assess and guide CVD risk management. Currently, early control of the HIV disease activity and managing the risk factors for CVD might partially modulate the immune dysfunction and subsequently reduce cardiovascular complications.

Initiation and maintaining HIV viral suppression through ART are crucial for prevention of CVD. In spite of the increased risk of CVD in the population receiving ART, it is known from D:A:D study that the absolute risk is small and the benefits of ART outweigh the risks [43]. Also, SMART study concluded that after one month of stopping ART, there is increased level of D-dimer and IL-6 with associated high risk for all-cause mortality [25]. However, changing ART regimen from PIs to nonnucleoside reverse transcriptase inhibitors (NNRTIs) may improve the lipid profile by increasing HDL cholesterol levels [57]. There are several barriers governing ART regimen selection such as economic burden of the drugs, limited availability of laboratory monitoring, and individual patient management. For instance, in Africa the cost of first line treatment is around $175 per year; the costs of the second line drugs can be ten times higher. In addition, effectiveness depends on high levels of adherence (at least 85 to 90 percent), for which counseling and follow-up to ensure adherence are required [58]. In conclusion while selection of ART regimen depends on the individual CVD risk and the duration of ART exposure, it might not be suitable strategy in low income countries to adopt certain ART regimens such as NNRTIs. Nevertheless, frequent monitoring of traditional risk factors such as dyslipidemia might help overcoming such a challenge [59].

Assessment of CVD risk for ART population is fundamental to guide risk management. However, utilizing conventional tools such as Framingham equation might inaccurately estimate the CVD risk. That is mainly because Framingham equation is used for non-HIV individuals and predicts the risk over a relatively long period. As previously mentioned HIV-infected individuals have significant CVD risk by 6 years' period [43]. Recently, several cardiovascular risk equations have been developed for HIV-positive patients. In a European multicenter study, conducted on 22,625 HIV-infected patients, an HIV specific model was able to accurately predict CVD better than conventional risk prediction models [60]. Thus, utilizing HIV specific model incorporating both routine CVD risk parameters and exposure to individual ART is useful in estimating CVD risks in HIV-infected persons compared with conventional risk prediction models [60]. The currently available recommendations for screening for the presence of CVD risk factors in persons with HIV infection take into account the evidence for dyslipidemia, insulin resistance, and changes in body fat distribution that have been shown to occur with HAART [61]. Nevertheless, referral for diagnostic testing should be assessed in light of the underlying disease or any comorbidity that might limit the life expectancy of the HIV patient [62].

To date, there is no evidence to suggest that HIV-positive patients need to be offered more aggressive management of dyslipidemia than those used in the general population. In 2013, the American College of Cardiology (ACC)/American Heart Association (AHA) issued updated practice guidelines for the treatment of blood cholesterol to reduce atherosclerotic CVD risk in adults [63, 64]. These guidelines recommend offering statin therapy of different intensity based on an individual's absolute risk (new calculator of risk provided in the recommendations) rather than aiming for a specific low-density lipoprotein (LDL) target level [64]. Accordingly, patients with the highest CVD risk are treated with high intensity statins and both primary and secondary prevention are addressed [63, 64]. Selected lipid-lowering drugs, such as pravastatin or atorvastatin, appear to be safely used in ART-treated patients [65]. Also, statins are known to have anti-inflammatory effects that are particularly beneficial to CVD [66]. In Intervention Trial Evaluating Rosuvastatin (JUPITER) study, anti-inflammatory treatment with rosuvastatin statistically significantly reduced mortality and risk of venous thrombotic disease in apparently healthy subjects with elevated hs-CRP (>2 mg/dL) and “normal” LDL cholesterol (<130 mg/dL) [67].

The prevalence of hypertension in HIV population has not been established with certainty [68]; however, hypertension remains a powerful predictor of CVD events as in the general population [69]. The prevalence of hypertension is expected to increase with improved survival of HIV patients and increased prevalence of HIV among patients in certain high risk ethnic subgroups [70]. Guidelines for the effective diagnosis and management of hypertension in non-HIV patients should be applied to patients with HIV until further data are available [68]. Also, impaired glucose tolerance is increased in patients with HIV and is associated with ART exposure [71]. In patients with HIV, intensive lifestyle intervention, metformin, and thiazolidinediones tend to reduce insulin resistance; however, the long-term effectiveness of these agents for the prevention and treatment of diabetes mellitus in patients with HIV is not known [68, 72]. Patients initiating ART should be screened for impaired fasting glucose and diabetes mellitus by measurement of fasting glucose levels or hemoglobin A1C levels at baseline, annually, and after changes are made to ART regimens [68].

Smoking is a classic risk factor for CVD, and in the general population, the risk of coronary heart disease and mortality considerably reduced within the first 2 years of stopping smoking [73]. Data from HIV-infected subjects in the D:A:D study showed that cessation of smoking decreases the risk of CVD, with increasing years of having stopped smoking [74]. In addition to obstacles to cessation known for the general population, substance abuse, psychiatric disorders, low socioeconomic status, poor access to care, and resulting low utilization of cessation programs are more prevalent with HIV and present significant risks for continued smoking and barriers to cessation [75].

There are several small studies conducted to address the impact of smoking cessation programs on HIV population [76–79]. One randomized clinical trial comparing a program of nicotine replacement therapy (NRT), self-help materials, and phone counseling with a usual care program comprised only of self-help materials and NRT found that HIV-infected smokers in the phone counseling group had abstinence rates of 36.8%, compared with 10.3% in the usual care group [80]. Limited data are available regarding pharmacologic therapy other than NRT for smoking cessation in HIV-infected populations. Nevertheless, there are several potential interactions between ART and smoking cessation pharmacotherapy. For example, ritonavir combined with lopinavir can significantly decrease plasma concentrations of bupropion [81]. It is essential to conduct more aggressive interventions to increase the efficacy and generality of smoking cessation programs in HIV-positive patients.

## 8. Conclusion

As HIV-positive patients live longer CVD risk is increasing. The risk of CVD in HIV-positive patients is a complex mix of the traditional cardiovascular risk factors and nontraditional risk factors such as immune dysfunction. The evaluation of an individual's CVD risk should be assessed routinely, and the prevention of CVD should therefore be considered in terms of the patient's overall CVD risk and HIV disease stage. Early initiation of ART, close monitoring of the drugs toxicity, and ensuring high level of adherence are fundamental for CVD risk modification. In addition, control of traditional risk factors such as smoking and dyslipidemia is greatly needed for HIV-positive population.

## Figures and Tables

**Table 1:** Factors that might confer an increased risk of cardiovascular diseases in HIV patients.

Traditional	Nontraditional
Age	Systemic inflammation
Smoking	Low CD4^+^ T-cells count
Obesity	Elevated C-reactive protein (CRP)
Diabetes mellitus	Elevated interleukin-6 (IL-6)
Hypertension	Elevated D-dimer
HIV-associated lipodystrophy syndrome (HALS)	Elevated HIV RNA level
	Role of drugs [protease inhibitors (PIs)]

## Abbreviations

HIV: Human immunodeficiency virus
ART: Antiretroviral therapy
CVD: Cardiovascular disease
MI: Myocardial infarction
HALS: HIV-associated lipodystrophy syndrome
CRP: C-reactive protein
IL-6: Interleukin-6
CMV: Cytomegalovirus
PIs: Protease inhibitors
NNRTIs: Nonnucleoside reverse transcriptase inhibitors
NRT: Nicotine replacement therapy.
